# Integrated Management of *Meloidogyne Incognita* and Soilborne Fungi Infecting Cucumber Under Protected Cultivation

**DOI:** 10.2478/jofnem-2022-0042

**Published:** 2022-11-20

**Authors:** J. A. Patil, Saroj Yadav, Sewak Ram, Anil Kumar, Satish Kumar

**Affiliations:** 1 Department of Nematology, Department of Plant Pathology, CCS Haryana Agricultural University, Haryana, Hisar, India

**Keywords:** *Cucumis sativus*, fungus, integration, management, *Meloidogyne incognita*, *Purpureocillium lilacinum*

## Abstract

Relative efficacy of various approaches for management of *Meloidogyne incognita* and the soilborne fungus *Fusarium oxysporum* f. sp. *cucumerinum* has been tested in cucumber under protected cultivation conditions for two seasons. Management practices, namely, chemicals (fumigant, nonfumigant, and fungicide), organic amendments (neem cake, leaves, and oil opted as soil and seed treatment), and biocontrol agents (egg-parasitic fungus and *Purpureocillium lilacinum*), were combined for the management of the disease complex in a randomized block design. Two significant parameters were measured: plant growth parameters (shoot length, dry shoot weight, dry root weight, and yield) and disease parameters (galls per plant, final nematode population, egg masses per plant, and fungal incidence). All treatments significantly improved plant growth parameters and reduced nematode reproduction as compared to untreated check. The integration of formalin and neem oil seed treatment favors the low root galling index compared to all other treatments in both the seasons. Formalin and neem oil seed treatment reduced the nematode population and fungal incidence, and increased the yield of cucumber during both the seasons.

Cucumber (*Cucumis sativus* L.) is the most widely cultivated crop under protected conditions in the world. Cucumber is a good source of minerals, vitamins, and fibers (Weng and Sun, 2011). Continuous monoculture at ideal temperature and relative humidity under polyhouse conditions favors nematodes and fungal diseases (Minuto *et al*., 2006; Patil *et al*., 2017b). Synergistic interaction between fungal pathogens and root-knot nematode causes heavy losses to the host crop (Ragozzino and D’Errico, 2012; Patil *et al*., 2018b). Severe damping off and nematode disease complex symptoms have been reported in cucumber-cultivating regions under polyhouse conditions (Greco and Esmenjaud, 2004; Katan, 2017; Patil *et al*., 2017b). Severity of damping off may vary depending on plant variety, environment, and soil texture. Soil temperature (up to 20–25oC) plays a significant role in damping off disease due to *Fusarium oxysporum*.

Root-knot nematode (*Meloidogyne incognita*) is a highly damaging nematode under protected cultivation conditions (Koenning *et al*., 1999; Greco and Esmenjaud, 2004; Sharma *et al*., 2007; Patil, 2017a; Murungi *et al*., 2018). In Haryana (India), the root-knot nematode frequency of occurrence was 63.15% reported under protected conditions (Patil, 2017a). Plants infected by root-knot nematode show typical galling on roots and express symptoms similar to those caused by nitrogen deficiency (Good, 1968; Kepenekci *et al*., 2016). Management of *Meloidogyne* spp. is very difficult due to their wide host-parasitizing ability, short life cycle (within20–25 d), high reproduction potential, and sedentary endoparasitic nature (Mauchline *et al*., 2004). The chemicals applied to crops are not always effective, and overuse causes serious bionomic problems (Fàbrega *et al*., 2013). Therefore, new alternative methods have been used to manage *Meloidogyne* spp. and fungal pathogens in polyhouse conditions, including volatile and nonvolatile nematicides, fungicides, organic amendments, and biocontrol strategies (Timper, 2011; Abd-Elgawad and Askary, 2018). Root-knot nematode is difficult to manage with only one technique (Barker *et al*., 1985); therefore, integrated management approaches have good potential against this notorious pest. Excellent and extensive research work has been conducted to manage root-knot nematode and soilborne fungal pathogens affecting vegetable crops under protected conditions, including soil solarization (Kumar *et al*., 2019); soil fumigation (Patil *et al*., 2018a, 2018b); and use of organic amendments (Patil, 2017a; Patil *et al*., 2017c) like biocontrol agents (Collange *et al*., 2011; Mcsorley, 2011), neem products such as neem oil, neem cake, and neem leaves (Yadav *et al*., 2018, 2021); and bioagents like *Paecilomyces lilacinus* (Patil *et al*., 2021)

However, a little work has been carried out for management of the disease complex in cucumber under protected cultivation conditions. The aim of this study was to examine the integrated management strategies against *M. incognita* and fungal pathogens in cucumber under polyhouse conditions.

## Materials and Methods

Experiments were conducted in naturally infested polyhouse with both pathogens under protected cultivation conditions (naturally ventilated polyhouse, 200 microns of transparent polyethylene sheet) on cucumber during 15 April to 19 July 2016 and 25 August to 5 December 2016 at the Department of Horticulture, CCS HAU, Hisar, Haryana, India (latitude: 29°10¢N, longitude: 75°46¢E, and altitude: 215.2 m). Effective treatments were selected from previous studies, where a large number of chemicals (Patil *et al*., 2018a; Kumar *et al*., 2019), organic amendments (Patil, 2017a; Patil *et al*., 2017c, 2018c, 2020a), and bioagents (Patil *et al*., 2018b, 2021) were tested, as given in Table 1.

**Table 1:** Treatment details of integrated approaches evaluated on cucumber against root-knot nematode and *Fusarium* in polyhouse conditions.

S. No.	Treatments
1	Neem cake 200 g/m^2^ + neem oil seed treatment 20% v/w
2	Neem cake 200 g/m^2^ + *Purpureocillium lilacinum* seed treatment 20 g/kg seed
3	Neem leaves 200 g/m^2^ + neem oil seed treatment 20% v/w
4	Neem leaves 200 g/m^2^ + *P. lilacinum* seed treatment 20 g/kg seed
5	*P. lilacinum* soil application 50 g/m^2^ + neem oil seed treatment 20% v/w
6	*P. lilacinum* soil application 50 g/m^2^ + *P. lilacinum* seed treatment 20 g/kg seed
7	Formalin 250 ml/m^2^ + *P. lilacinum* seed treatment 20 g/kg seed
8	Formalin 250 ml/m^2^ + neem oil seed treatment 20%v/w
9	Carbofuran 10 g/m^2^
10	Bavistin 2 g/l water
11	Control (inoculated)

## Organic amendment and bioagent

A commercial bio-product containing *Purpureocilliumlilacinum* (1% W.P.) (CFU of 2 × 106/g) was procured from the IIHR, Bengaluru. Neem leaves were first chopped into small pieces with the help of scissors or a grinder. Neem cake and oil were procured from the local market. Products and their application details are described in Table 2.

**Table 2:** Integrated management practices and their application methods, time, and doses used in cucumber against root-knot nematode and *Fusarium* in polyhouse conditions.

S. No.	Management strategies	Management method	Material used	Application methods	Time of application	Doses
1	Chemical	Fumigant	Formalin	Fumigation	20 days before sowing	30%, i.e., 250 ml/m^2^
		Nonfumigant	Carbofuran	Soil application	Before sowing	10 g/m^2^
		Fungicide	Bavistin	Drenching	Before sowing	2 g/l water
2	Biological	Egg-parasitic fungus	*Purpureocillium lilacinum*	Soil application	15 days before sowing	50 g/m^2^
				Seed treatment	6 hours before	20 g/kg
					sowing	seed
3	Cultural	Organic amendment	Neem cake	Soil application	15 days before sowing	200 g/m^2^
			Neem leaves	Soil application	15 days before sowing	200 g/m^2^
			Neem oil	Seed treatment	6 hours before sowing	20% v/w
4	Check	-	-	-		Untreated infested

### Field preparation

Experiments were carried out in two consecutive crop seasons at the polyhouse field (sand, silt, and clay, 79.7%, 11.4%, and 9.0%, respectively) naturally infested with root-knot nematode. *Meloidogyne* species were identified by perineal patterns (Netscher and Taylor, 1974). Fungus isolation from infected plant roots was carried out using potato dextrose agar (PDA), and the isolated fungal species was identified as *F. oxysporum* on the basis of their morphological characteristics (Leslie and Summerell, 2006).

### Application method

Harrowing was carried out to maintain the porosity of the field before application of various treatments.Fumigation with formalin was performed before 20 d of sowing, and other chemicals were incorporated at the sowing time. Drenching of formalin was carried out, and the soil was covered with transparent polyethene sheet (LLDP 25 mm). Carbofuran granules were directly applied by using the broadcasting method, and fungicide solution was prepared by mixing 2 g bavistin in 1 L water and drenched. Neem cake and neem leaves were applied before sowing, and seed treatment with *P. lilacinum* was carried out before sowing for 6 hours. After the treatment, the seeds were dried in shade for 6 hr.

### Experimental design

Integrated management strategies were evaluated against *M. incognita* and *F. oxysporum* in cucumber under polyhouse conditions for two consecutive seasons. The initial nematode was 256 J_2_/200 cm^3^ and 291 J_2_/200 cm^3^ soil during the first and second seasons, respectively. A total of 33 plots (20 × 1 m^2^ each) was measured, and all treatments were replicated thrice in a randomized block design. Each bed acts as single replication (44 plants per replication) of the treatment. Row-to-row and plant-to-plant spacings (60 × 45 cm) were maintained. Three seeds of cucumber (cv. Sania, susceptible to both the pathogens) were sown at each place on beds, and after germination, one plant was maintained. General care and maintenance of plants were undertaken as recommended by CCS Haryana Agricultural University, Hisar (Anonymous, 2016). The plants were supported by jute thread, and 0.1% azadirachtin sprays were applied to protect the crop from whitefly.

### Data collection and statistical analysis

At harvesting, plant parameters such as shoot length and dry root weight were measured (five plants per plot), and the cumulative yield of cucumber has been determined by adding all picked yield. On each harvest date, marketable cucumber fruits in each plot were picked and weighed. Five subsamples were collected (15–20 cm depth) from each replication, and 200 cm^3^ composite sample was assessed by using Cobb’s method (Cobb, 1918), followed by modified Baermann’s funnel technique (Schindler, 1961) for estimation of final nematode populations. Second-stage juveniles (*M. incognita*) were counted under a binocular microscope by the dilution method (Hooper, 1986).

Fungal incidence of *Fusarium* was recorded from five plants per plot at 15 d and 30 d after sowing. Conferring to a 0 to 5 scale (0 = root healthy; 1 = 1–10% affected root surface (a.r.s.); 2 = 11–25% a.r.s.; 3 = 26–50% a.r.s.; 4 = 51–75% a.r.s.; and 5 ≥76% a.r.s.). Analysis of *F. oxysporum* infection was confirmed by isolation of the fungus. Consequently, symptomatic tissues of cucumber plants (1 mm^2^) were sterilized, rinsed in sterile distilled water, placed in petri dishes containing acidified PDA medium amended with sodium hypochloride (0.1%), and assessed using the following formula:


$$\text{ Disease incidence }=\frac{\text{ No. of infected plants }\times 100}{\text{ Total no. of plant assessed }}$$

All data were subjected to analysis of variance (ANOVA) using SPSS software to determine significant differences (*P* < 0.05) between treatments. Means were separated and compared using Duncan’s multiple range test. Differences in mean values were considered significant when *P* < 0.05. All of the experiments were repeated at least two times, with similar results.

## Results

### Impact of combined approaches on plant growth and yield of cucumber

**Figure 1:**
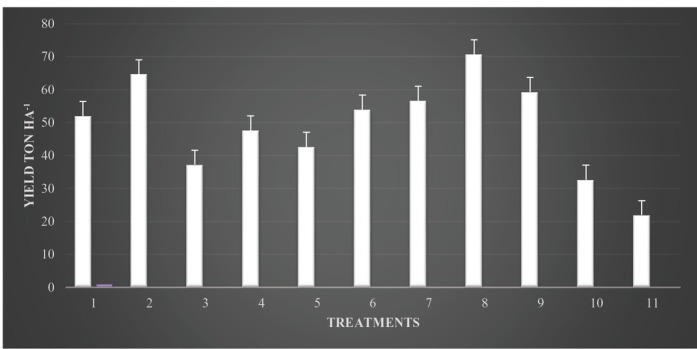
Effects of integrated management approaches on cucumber yield (ton per ha) against root-knot nematode and *Fusarium* in the first season in polyhouse conditions.Note: 1 = neem cake 200 g/m^2^ + neem oil seed treatment 20% v/w; 2 = neem cake 200 g/m^2^ + *Purpureocillium lilacinum* seed treatment 20 g/kg seed; 3 = neem leaves 200 g/m^2^ + neem oil seed treatment 20% v/w; 4 = neem leaves 200 g/m^2^ + *P. lilacinum* seed treatment 20 g/kg seed; 5 = *P. lilacinum* soil application 50 g/m^2^ + neem oil seed treatment 20% v/w; 6 = *P. lilacinum* soil application 50 g/m^2^ + *P. lilacinum* seed treatment 20 g/kg seed; 7 = formalin 250 ml/m^2^ + *P. lilacinum* seed treatment 20 g/kg seed; 8 = formalin 250 ml/m^2^ + neem oil seed treatment 20%v/w; 9 = carbofuran 10 g/m^2^; 10 = bavistin 2 g/l water; 11 = control (inoculated).

**Figure 2:**
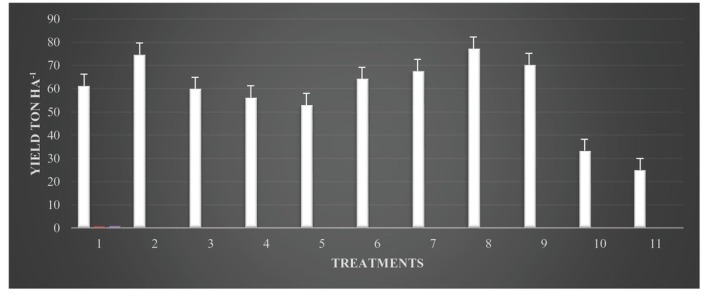
Effects of integrated management approaches on cucumber yield (ton per ha) against root-knot nematode and *Fusarium* in the second season in polyhouse conditions. Note: 1 = neem cake 200 g/m^2^ + neem oil seed treatment 20% v/w; 2 = neem cake 200 g/m^2^ + *Purpureocillium lilacinum* seed treatment 20 g/kg seed; 3 = neem leaves 200 g/m^2^ + neem oil seed treatment 20% v/w; 4 = neem leaves 200 g/m^2^ + *P. lilacinum* seed treatment 20 g/kg seed; 5 = *P. lilacinum* soil application 50 g/m^2^ + neem oil seed treatment 20% v/w; 6 = *P. lilacinum* soil application 50 g/m^2^ + *P. lilacinum* seed treatment 20 g/kg seed; 7 = formalin 250 ml/m^2^ + *P. lilacinum* seed treatment 20 g/kg seed; 8 = formalin 250 ml/m^2^ + neem oil seed treatment 20%v/w; 9 = carbofuran 10 g/m^2^; 10 = bavistin 2 g/l water; 11 = control (inoculated).

No phytotoxic effect was observed in formalin-treated plots. During both the seasons, significantly (*P* < 0.05) highest shoot length, dry shoot weight, and dry root weight of cucumber were obtained by application of formalin with neem oil seed treatment as compared to other treatments (Table 3). In the first season (Fig. 1), quantity (yield) of cucumber fruit was less in the untreated control (22.0 t/ha), while the collective yield significantly (*P* < 0.05) enhanced in treated plots (70.7 t/ha) (Table 3). In the second season, effects of combined methods on production variables were much more pronounced than those in the earlier season. However, an analogous trend was found in the second season experiment, and plant height (186.6 cm) was significantly greater than that in other treatment. The average yield was significantly higher (Fig. 2) with formalin and neem oil seed treatment (77.3 t/ha) than that in the untreated check (25.0 t/ ha) (Table 3). However, the cumulative fruit yield was significantly (*P* < 0.05) higher in all treated plots, including those treated with carbofuran and bavistin, than in the untreated control.

**Table 3:** Effect of integrated management practices on growth and yield of cucumber against root-knot nematode and *Fusarium* in polyhouse conditions.

Sr. no.	Treatments	First season			Second season			
		
		Shoot length (cm)	Dry shoot weight (g)	Dry root weight (g)	Yield (t/ha)	Shoot length (cm)	Dry root weight (g)	Yield (t/ha)
1	Neem cake 200 g/ m^2^ + neem oil seed treatment 20% v/w	176.4^e,f^	26.7^b,c,d^	8.58^b,c,d^	52.0^f^	172.7^e,f^	9.00^d,e^	61.3^e^
2	Neem cake 200 g/ m^2^ + *Purpureocillium lilacinum* seed treatment 20 g/kg seed	180.6^f,g^	35.4^e^	11.58^c,d^	64.7^i^	184.0^h^	14.33^g,h^	74.7^h,i^
3	Neem leaves 200 g/ m^2^ + neem oil seed treatment 20% v/w	160.4^c^	21.6^b^	7.52^b,c^	37.3^c^	155.0^c^	7.00^b,c^	60.0^d,e^
4	Neem leaves 200 g/ m^2^ + *P. lilacinum* seed treatment 20 g/kg seed	170.2^d,e^	24.6^b,c^	8.26^b,c^	47.7^e^	167.3^d,e^	8.30^c,d^	56.3^c,d^
5	*P. lilacinum* soil application 50 g/ m^2^ + neem oil seed treatment 20% v/w	163.2^c,d^	22.9^b,c^	8.11^b,c^	42.7^d^	161.7^c,d^	7.67^b,c,d^	53.0^c^
6	*P. lilacinum* soil application 50 g/m^2^ + *P. lilacinum* seed treatment 20 g/kg seed	176.5^e,f^	28.6^c,d^	8.51^b,c,d^	54.0^f,g^	174.7^f,g^	10.33^e,f^	64.3^e,f^
7	Formalin 250 ml/m^2^ + *P. lilacinum* seed treatment 20 g/kg seed	179.6^e,f,g^	31.6^d,e^	9.58^b,c,d^	56.7^g,h^	180.0^g,h^	11.00^f^	67.7^f,g^
8	Formalin 250 ml/ m^2^ + neem oil seed treatment 20%v/w	186.6 ^g^	36.7^e^	12.58^d^	70.7^j^	197.9^i^	15.67^h^	77.3^i^
9	Carbofuran 10 g/m^2^	177.9^e,f,g^	33.2^d,e^	10.58^b,c,d^	59.3^h^	181.7^g,h^	13.00^g^	70.3^g,h^
10	Bavistin 2 g/l water	128.0^b^	22.6^b,c^	6.58^b^	32.7^a^	132.0^b^	6.00^b^	33.3^b^
11	Control (inoculated)	107.2^a^	7.4^a^	2.58^a^	22.0^b^	107.2^a^	2.91^a^	25.0^a^

Note: Data are means of three replications. In each column, values with the same letters denote a nonsignificant difference (*P* < 0.05) according to Duncan’s test of multiple comparisons in a randomized block design.

### Impact of combined approaches on nematode reproduction and fungal incidence in cucumber

Data indicated that (Table 4) during the first season (2015–2016), the final nematode population (155 J_2_ 200 cm^3^ × soil^-1^) and galls per plant were significantly reduced with formalin and neem oil compared to untreated inoculated check. Application of formalin and neem oil seed treatment was most effective in reducing root galling, nematode population, and reproduction factor. In the second season (2016– 2017), the final nematode population and galls per plant had declined more than those in the first season. An analogous result was found in 2016–2017 experiment on nematode disease parameters such as final nematode population, gall per plants, and reproduction factor. Significantly reduced galls were observed with formalin + neem oil seed treatment, followed by formalin and *P. lilacinum* seed treatment, compared to untreated check.

The severity of the disease complex was reduced in the treated plots in both the seasons as compared to untreated plots at 15 d and 30 d after sowing (Table 5). Fungal incidence and root galling were severe in the untreated plots in the first crop season. In the second season of crop, a significant reduction in fungal incidence was found in formalin and neem oil seed-treated plots as compared to the untreated control (Table 5) at 15 d and 30 d after sowing. The reduced galling echoed a decrease in fungal disease incidence recorded in the second season of cucumber. Fungal incidence was significantly lowest in both the seasons wherever formalin and neem oil seed treatment was applied, followed by formalin and *P. lilacinum* seed treatment, as compared to untreated inoculated check.

**Table 4:** Effect of integrated management practices on the nematode population on cucumber infested with root-knot nematode and *Fusarium* under polyhouse conditions.

Sr.No.	Treatments	First season				Second season			
		
		Egg masses per plant	Galls per plant	Final nematode population 200 cm^3^ soil^-1^	Reproduction factor	Egg masses per plant	Galls per plant	Final nematode population 200 cm3 soil^-1^	Reproduction factor
1	Neem cake 200 g/m^2^ + neem oil seed treatment 20% v/w	185^e^	210^f^	250^e^	0.8	166^f^	203^e^	238^e^	0.9
2	Neem cake 200 g/m^2^ + *Purpureocillium lilacinum* seed treatment 20 g/kg seed	95^b^	112^C^'	217^C^	0.7	85^c^	103^c^	201^c^	0.8
3	Neem leaves 200 g/m^2^2 + neem oil seed treatment 20% v/w	224^g^	241^h^	340^h^	1.2	217^h^	230^h^	328^h^	1.3
4	Neem leaves 200 g/m^2^ + *P. lilacinum* seed treatment 20 g/kg seed	195^f^	220^f,g^	275^f^	0.9	183^g^	214^f^	262^f^	1.0
5	*P. lilacinum* soil application 50 g/m^2^ + neem oil seed treatment 20% v/w	215^g^	230^g^	315^g^	1.0	208^h^	221^g^	312^g^	1.2
6	*P. lilacinum* soil application 50 g/ m^2^ + *P. lilacinum* seed treatment 20 g/kg seed	160^d^	198^e^	238^d^	0.8	145^e^	181^d^	233^e^	0.9
*7*	Formalin 250 ml/ m^2^ + *P. lilacinum* seed treatment 20 g/kg seed	55^a^	75b	170b	0.5	48^b^	73^b^	161^b^	0.6
8	Formalin 250 ml/ m^2^ + neem oil seed treatment 20%v/w	48^a^	60^a^	155^a^	0.5	32^a^	53^a^	141^a^	0.5
9	Carbofuran 10 g/m^2^	145^c^	185^c^	218^c^	0.7	126d	180c	214d	0.8
10	Bavistin 2 g/l water	456^h^	485^i^	718^i^	2.4	446^i^	482^i^	696^i^	2.7
11	Control (inoculated)	553^i^	570^i^	890^i^	3.0	560^i^	594^i^	902^i^	3.5

Note: Data are means of three replications. In each column, values with the same letters denote a nonsignificant difference (*P* < 0.05) according to Duncan’s test of multiple comparisons in a randomized block design.

**Table 5:** Effect of integrated management practices on fungal incidence on cucumber infested with root-knot nematode and *Fusarium* under polyhouse conditions.

Sr. No.	Treatments	Percent fungal incidence			
		First season		Second season	
		
		15 d after	30 d after	15 d after	30 d after
		sowing	sowing	sowing	sowing
1	Neem cake 200 g/m^2 +^ neem oil seed treatment 20% v/w	13^a,b,c^	20^b,c^	27^a^	33^c,d^
2	Neem cake 200 g/m^2^ + *Purpureocillium lilacinum* seed treatment 20 g/kg seed	13^a,b,c^	13^a,b^	27^a^	20^b,c^
3	Neem leaves 200 g/m^2^ + neem oil seed treatment 20% v/w	20^b,c^	20^b,c,d^	33^a^	33^c,d^
4	Neem leaves 200 g/m^2^ + *P. lilacinum* seed treatment 20 g/kg seed	20^a,b,c^	20^b,c^	27^a^	20^b,c^
5	*P. lilacinum* soil application 50 g/m^2^ + neem oil seed treatment 20% v/w	20^b,c^	27^b,c,d^	33^a^	40^d,e^
6	*P. lilacinum* soil application 50 g/m^2^ + *P. lilacinum* seed treatment 20 g/kg seed	27^b,c^	33^c,d^	33^a^	40^d,e^,
7	Formalin 250 ml/m^2^ + *P. lilacinum* seed treatment 20 g/kg seed	7^a,b^	7^a,b^	20^a^	14^a,b^
8	Formalin 250 ml/m^2^ + neem oil seed treatment 20%v/w	1^a^	1^a^	14^a^	1^a^
9	Carbofuran 10 g/m^2^	27^b,c^	33^d^	33^a^	53^e^
10	Bavistin 2 g/l water	33^b,c^	40^c,d^	33^a^	40^d,e^
11	Control (inoculated)	47^c^	80^e^	40^a^	93^f^

Note: Data are means of three replications. In each column, values with the same letters denote a nonsignificant difference (*P* < 0.05) according to Duncan’s test of multiple comparisons in a randomized block design.

## Discussion

Root-knot nematodes and soilborne fungi are the main constraints in the production of vegetable crops under polyhouse conditions including cucumber throughout India, with few effective control methods available (Collange *et al*., 2011; Jones, 2017). Therefore, it is imperative requirement toward discovery actual and economically practicable fumigant nematicides for management of *M. incognita* and soilborne fungi. In this experiment, we demonstrated that integrated use of formalin and seed treatment with neem oil was highly effective against *M. incognita* and soilborne fungi and significantly enhanced cucumber yield. Application of formalin and neem oil seed treatment suppressed the nematode population and reproduction rate of *M. incognita* during both the seasons, and neem oil was also helpful in enhancing the germination percentage. Similar findings were reported by Kumar *et al*. (2019), and Patil *et al*. (2020a). Combined application of formalin and seed treatment with neem oil suppressed soilborne fungal infection in cucumber during both the seasons. After the formalin treatment, soil was quickly covered with polythene sheet (LLDP); this was very helpful in enhancing the efficacy of formalin in the form of fumes. The covered polythene sheet was highly beneficial in modification of physicochemical and biological properties like increasing the availability of mineral nutrients and soluble organic matter, which affects soil microflora and fauna (Mola *et al*., 2021).

Damage caused by *M. incognita* along with *F. oxysporum* f. sp. *cucumerinum* has adverse effects on production of cucumber in polyhouse conditions, causing significant monetary losses to polyhouse farmers (Koenning *et al*., 1999; Patil *et al*., 2017b). This is possibly because juveniles of root-knot nematode puncture the roots, through fungal penetration. The achievements also concord with previous studies (Stephan *et al*., 1998; Meher *et al*., 2010; Patil *et al*., 2018c; Kumar *et al*., 2019), which showed that fumigants were effective in reducing nematode populations and significantly increased the growth and yield of vegetables. Based on our findings, integrated management practices, that is, formalin along with neem oil seed treatment, have been reported to enhance cucumber yield, decline in fungal incidence, and significantly reduce the nematode population. This practice could be highly beneficial to farmers for the vegetable production under polyhouse cultivation conditions. These findings concur with previous studies (Akhtar and Malik, 2000; Collange *et al*., 2011; Faruk *et al*., 2011; Katan, 2017) which reported that integrated management has been used to manage *M. incognita* and fungal disease complex in vegetable crops. Fumigation and seed treatment with organic oils have been widely suggested to control soilborne pathogens by various researchers (Moubark and Abdel-Monaim, 2011; Radwan *et al*., 2012; Patil *et al*., 2018c, 2020a; Kumar *et al*., 2019). Nevertheless, information on the integrated management of both the pathogens under polyhouse conditions on cucumber growth and fruit yield is limited in India. Neem oil may have enhanced beneficial microbial activity, resulting in a significant improvement of the soil profile and germination percentage of cucumber seeds (Patil *et al*., 2020a).

In polyhouse trials, our findings also coincide with the study by Stephan *et al*. (1998) who stated that the organic amendments applied to chemicals reduced nematode reproduction in tomato and eggplant. The disease complex was minimum wherever soil was subjected to formalin and neem oil seed treatment, followed by application of formalin with *P. lilacinum*. These findings are in agreement with Akhtar *et al*. (2005), Patil (2017b), Mishra *et al*. (2017), and Patil *et al*. (2021) which showed that integration of fumigants along with organic oil reduced the disease complex severity than individual application of carbofuran and bavistin. Integration of neem oil with other approaches has been useful in increasing microbial activity of the soil and suppressing fungus and nematode reproduction (Oka *et al*., 2007; Oka, 2010; Moosavi, 2020). Biofumigation of neem leaves and brassica leaves was also found to increase the soil microflora and fauna activity and reduce the root-knot nematode population (Yadav *et al*., 2018; Patil *et al*., 2020b). During the first crop season, yield and growth of the cucumber were less than those in the second season due to the seasonal effect. The integrated use of fumigants and seed treatment with neem oil used in present investigation ensured suppressive effects on nematode and fungus. Although the use of formalin and organic oil enhanced the cucumber yield over control, the crop was infected by root-knot nematode and soilborne fungi. These integrated management strategies have been taken into deliberation by polyhouse growers for vegetables.
